# Case report: Novel genotype of ALG2-CDG and confirmation of the heptasaccharide glycan (NeuAc-Gal-GlcNAc-Man2-GlcNAc2) as a specific diagnostic biomarker

**DOI:** 10.3389/fgene.2024.1363558

**Published:** 2024-05-06

**Authors:** Ivan Martínez Duncker, Denisse Mata-Salgado, Ibrahim Shammas, Wasantha Ranatunga, Earnest James Paul Daniel, Mario E. Cruz Muñoz, Melania Abreu, Héctor Mora-Montes, Miao He, Eva Morava, Gildardo Zafra de la Rosa

**Affiliations:** ^1^ Laboratorio de Glicobiología Humana y Diagnóstico Molecular, Centro de Investigación en Dinámica Celular, Instituto de Investigación en Ciencias Básicas y Aplicadas, Universidad Autónoma del Estado de Morelos, Cuernavaca, Mexico; ^2^ Department of Clinical Genomics, Department of Laboratory Medicine and Pathology, Mayo Clinic, Rochester, MN, United States; ^3^ Palmieri Metabolic Disease Laboratory, Children’s Hospital of Philadelphia, Philadelphia, PA, United States; ^4^ Laboratorio de Inmunología Molecular, Facultad de Medicina, Universidad Autónoma del Estado de Morelos, Cuernavaca, Mexico; ^5^ Genos Médica, Ciudad de México, Mexico; ^6^ Departamento de Biología, División de Ciencias Naturales y Exactas, Campus Guanajuato, Universidad de Guanajuato, Guanajuato, Mexico; ^7^ Genetics Service, Cancer Center Tec100, Querétaro, Mexico

**Keywords:** CDG (congenital disorder of glycosylation), congenital myasthenic syndromes (CMS), ALG2, biomarker, glycan

## Abstract

This report outlines the case of a child affected by a type of congenital disorder of glycosylation (CDG) known as ALG2-CDG (OMIM 607906), presenting as a congenital myasthenic syndrome (CMS) caused by variants identified in *ALG2*, which encodes an α1,3-mannosyltransferase (EC 2.4.1.132) involved in the early steps of N-glycosylation. To date, fourteen cases of ALG2-CDG have been documented worldwide. From birth, the child experienced perinatal asphyxia, muscular weakness, feeding difficulties linked to an absence of the sucking reflex, congenital hip dislocation, and hypotonia. Over time, additional complications emerged, such as inspiratory stridor, gastroesophageal reflux, low intake, recurrent seizures, respiratory infections, an inability to maintain the head upright, and a global developmental delay. Whole genome sequencing (WGS) revealed the presence of two *ALG2* variants in compound heterozygosity: a novel variant c.1055_1056delinsTGA p.(Ser352Leufs*3) and a variant of uncertain significance (VUS) c.964C>A p.(Pro322Thr). Additional studies, including determination of carbohydrate-deficient transferrin (CDT) revealed a mild type I CDG pattern and the presence of an abnormal transferrin glycoform containing a linear heptasaccharide consisting of one sialic acid, one galactose, one N-acetyl-glucosamine, two mannoses and two N-acetylglucosamines (NeuAc-Gal-GlcNAc-Man2-GlcNAc2), ALG2-CDG diagnostic biomarker, confirming the pathogenicity of these variants.

## 1 Introduction

Congenital disorders of glycosylation (CDG) are an expanding group of hereditary metabolic disorders that represent genetic defects in the synthesis of glycans and their binding to proteins, lipids, and RNA (Chang et al., 2018; Ondruskova et al., 2021). Particularly, protein glycosylation is a process that can occur through *N*-linked glycosylation and/or *O*-linked glycosylation. Pathogenic variants in over 40 genes that participate in the *N*-glycosylation pathway clinically translate into multiple phenotypes, most of which include seizures, axial hypotonia, global developmental delay/intellectual disability, cerebellar atrophy, and delayed myelination (Cossins et al., 2013; Chang et al., 2018; Francisco et al., 2023). This highlights the crucial importance of *N*-glycosylated proteins for normal neuronal development and the establishment and maintenance of appropriate cognitive functions.

In addition to their neurological manifestations, *N*-glycosylation defects are also linked to neuromuscular disorders. Notable examples associated to congenital myasthenic syndromes (CMS) include pathogenic variants in *ALG2, ALG14*, *DPGAT1* and *GMPPB* (Belaya et al., 2015; Paprocka et al., 2021; Francisco et al., 2023). CMS are a heterogeneous group of hereditary disorders derived from alterations in signal transmission at the neuromuscular synapse, characterized by fatigable muscle weakness. ALG2-CDG (OMIM 607906), previously known as CDG-Ii, is an autosomal recessive rare disorder caused by pathogenic variants in *ALG2* (OMIM 607905) that encodes the α1,3-mannosyltransferase (EC 2.4.1.132) involved in the second and third steps of mannose addition during the early steps of *N*-glycosylation. Currently, it is recognized that ALG2-CDG exhibits a broad clinical spectrum, characterized primarily by global developmental delay and predominant muscular weakness. To date, 14 cases have been reported, including three patients from Latin America (Thiel et al., 2003; Cossins et al., 2013; Monies et al., 2014; Monies et al., 2016; Papazoglu et al., 2021; Ehrstedt et al., 2022). In addition to neurological and neuromuscular manifestations, abnormal coagulation, hepatomegaly, colobomas, and hearing loss have been reported (Thiel et al., 2003; Cossins et al., 2013; Monies et al., 2014; Monies et al., 2016; Papazoglu et al., 2021; Ehrstedt et al., 2022). This case report addresses the clinical findings and molecular diagnosis of an infant of Mexican ancestry affected by ALG2-CDG, supported by biochemical and genetic tests that evidence the deleterious effect of the identified heterozygous variants.

## 2 Case report and methods

The proband is a 1-year-old male of Mexican ancestry, born to non-consanguineous parents with no relevant family history of genetic alterations. He is the only child in the family, born by cesarean section at 40.1 weeks of gestation, with a birth weight of 2,995 kg (percentile 10-25), length of 48.5 cm (percentile 25-50), and head circumference of 35.4 cm (percentile 25-50). Apgar scores were 7 at 1 minute, improving to nine at 5 minutes, requiring 1 hour after birth of bag-mask resuscitation with two cycles and nasal prongs.

During the neonatal period, the child presented with inspiratory stridor, congenital hip dislocation, absence of crying, and drooling. Neurological evaluation revealed generalized hypotonia, proximal muscle weakness, decreased tendon reflexes, and the absence of both the Moro reflex and sucking reflex. Subsequently, additional symptoms emerged, including weak crying, both proximal and distal muscle weakness, gastroesophageal reflux, inability to maintain head control, recurrent infections, contractures in proximal joints, and feeding difficulties. Due to the latter, a gastrostomy was performed at 13 months, with continuous follow-up by the gastroenterology team.

Furthermore, at 1.5 months of age, the child experienced seizures characterized by absent gaze and brief cyanosis, which were partially controlled with valproic acid and levetiracetam.

In the current clinical assessment at 1 year and 7 months, the child weighs 8,350 g (percentile <3), has a head circumference of 44.8 cm (percentile <3), and a length of 79 cm (percentile <3 for expected height based on familial growth charts). Global developmental delay has been observed, accompanied by a poor response to painful stimuli, generalized muscle weakness, generalized hypotonia, depressed osteotendinous reflexes, laxity in distal joints, and seizures that are better controlled with valproic acid and lacosamide. It is important to note that these findings occur in the context of normal creatine kinase (CPK) levels.

**FIGURE 1:**
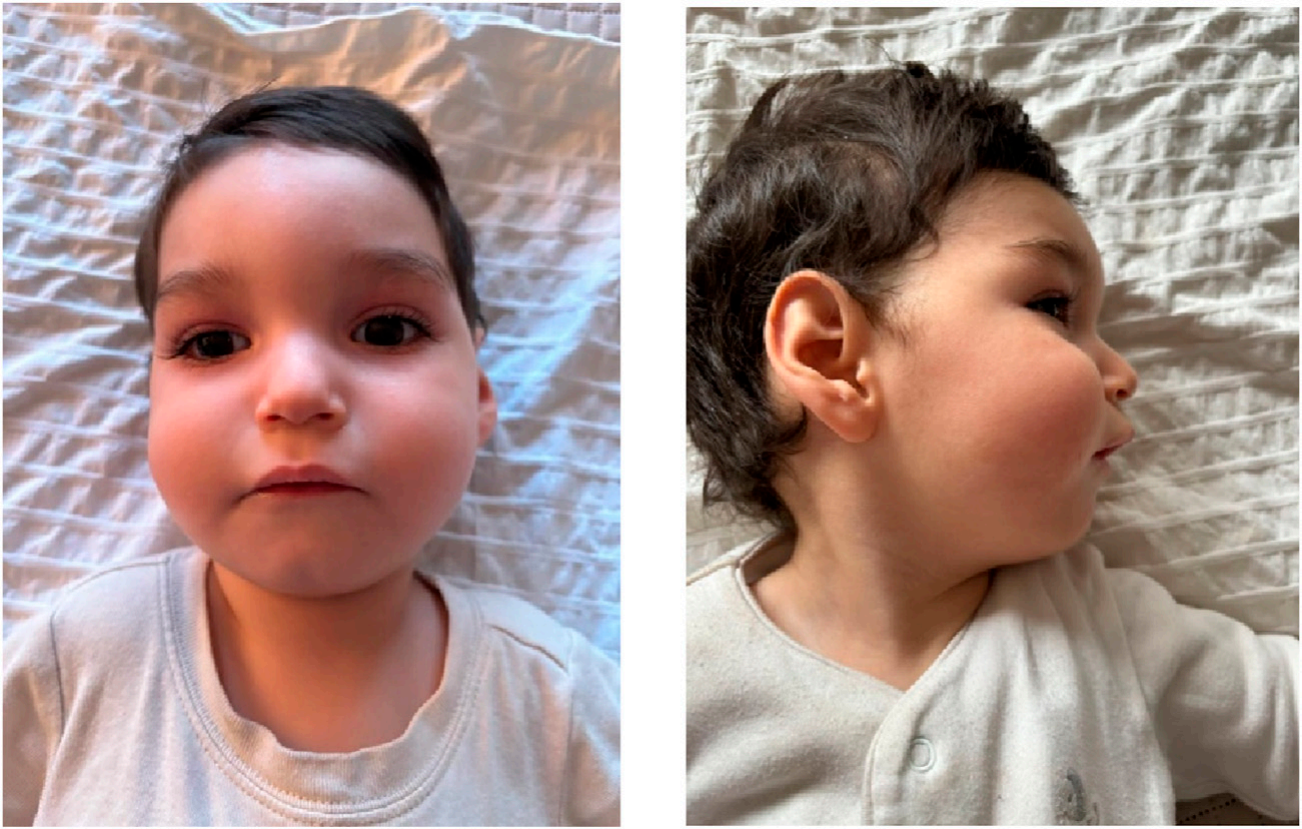
Dysmorphic facial features. Dysmorphic features include a prominent forehead, metopic prominence, fan-shaped eyebrows, long eyelashes, sunken eyes, nystagmus, wide nasal bridge, full cheeks, thick nasal tip, prominent upper lip, retrognathia, pointed ears with hypoplastic helix and prominent concha.

Dysmorphic features include a prominent forehead, metopic prominence, fan-shaped eyebrows, long eyelashes, sunken eyes, nystagmus, wide nasal bridge, full cheeks, thick nasal tip, prominent upper lip, high palate, retrognathia, pointed ears with hypoplastic helix and prominent concha (Figure 1), as well as wide-set nipples, dorsal hypertrichosis, and a congenital dermal melanocytosis (mongolian spot) over the sacral region, and non-communicating sacral dimple. Additionally, there are transverse creases on the hands, abduction of the first finger on the left hand due to a restrictive fold, mildly marked flexion creases in the phalanges, pads on the fingertips, and a thicker first toe on the left foot compared to the right. Brain imaging studies only revealed a temporal arachnoid cyst (17 × 9 mm). Renal ultrasound showed normal kidneys. The complete blood cell count, serum chemistry and coagulation tests were normal.

Genetic testing was initiated obtaining buccal samples for Whole Genome Sequencing (WGS) test by Biomegen (Querétaro, México). Libraries were sequenced from both ends in an Illumina platform reaching a depth of ∼30x. An internal bioinformatic process was applied that included alignment of the reading with the Human genome assembly GRCh37/hg19 and the Revised Cambridge Reference Sequence of the Human Mitochondrial DNA (NC_012920), variant call, annotation and exhaustive filtration of variants. The DRAGEN Copy Number Variant pipeline from Illumina was applied. All variants with an allelic frequency lower to 1% in the gnomAD database as well as the disease-causing variants annotated in HGMD and ClinVar were evaluated. Two variants in compound heterozygosity were identified in *ALG2*: NM_033087.4: c.1055_1056delinsTGA p.(Ser352Leufs*3) and NM_033087.4: c.964C>A p.(Pro322Thr). Parental testing revealed that the father carried the variant c.964C>A p.(Pro322Thr), while the mother carried the c.1055_1056delinsTGA p.(Ser352Leufs*3) variant. To date, there have been no reports of the occurrence of these variants in individuals affected by ALG2-CDG. Variant classification followed the guidelines of the American College of Medical Genetics (Richards et al., 2015).

The variant c.964C>A p.(Pro322Thr) is classified as a VUS (ClinVar 2155640; dbSNP rs1228242180), where proline, which is neutral and non-polar, is substituted by threonine, which is neutral and polar. Algorithms such as SIFT, PolyPhen-2, and Align-GVGD suggest that this variant is likely deleterious (Ng and Henikoff, 2003; Mathe et al., 2006; Tavtigian et al., 2006; Adzhubei et al., 2013). Additionally, STRUM was employed to investigate a potential alteration in protein stability induced by the amino acid substitution, measuring the differences in Gibbs free energy (ddG) between wild-type and mutated proteins (Quan et al., 2016). This analysis revealed a ddG of −0.5, suggesting a decrease in protein stability. Simultaneously, the predictive method SAAFEC-SEQ, supported by a decision tree machine learning algorithm, was employed (Li et al., 2021). This method utilizes physicochemical properties, sequence characteristics, and evolutionary information. A ddG of −1.07 was obtained, indicating a destabilizing effect predicted by this approach (Li et al., 2021). Interestingly, according to gnomAD exomes and genomes database (Chen et al., 2022) the variant is restricted to admixed American genetic ancestry group with a frequency of 0.00004572 or two in 43740 (gnomAD Exomes Version 4.0 as of December 2023). Interestingly, these alleles were found in one male and one female, indicating a heterozygous state.

As for the novel variant c.1055_1056delinsTGA p.(Ser352Leufs*3), it results in a premature stop codon, predicting truncation of the protein in the glycosyltransferase family one domain. The variant has the potential to impact protein function by altering the last 65 amino acids in the protein sequence. The variant is absent from gnomAD exomes and genomes database (gnomAD Exomes Version 4.0 as of December 2023). It is noteworthy that another variant p. (Ser352Tyrfs*2) is found in the same position resulting in the similar premature truncation of the protein. Interestingly, all 75 carriers of this allele are also heterozygous (dbSNP rs757837485).

**FIGURE 2:**
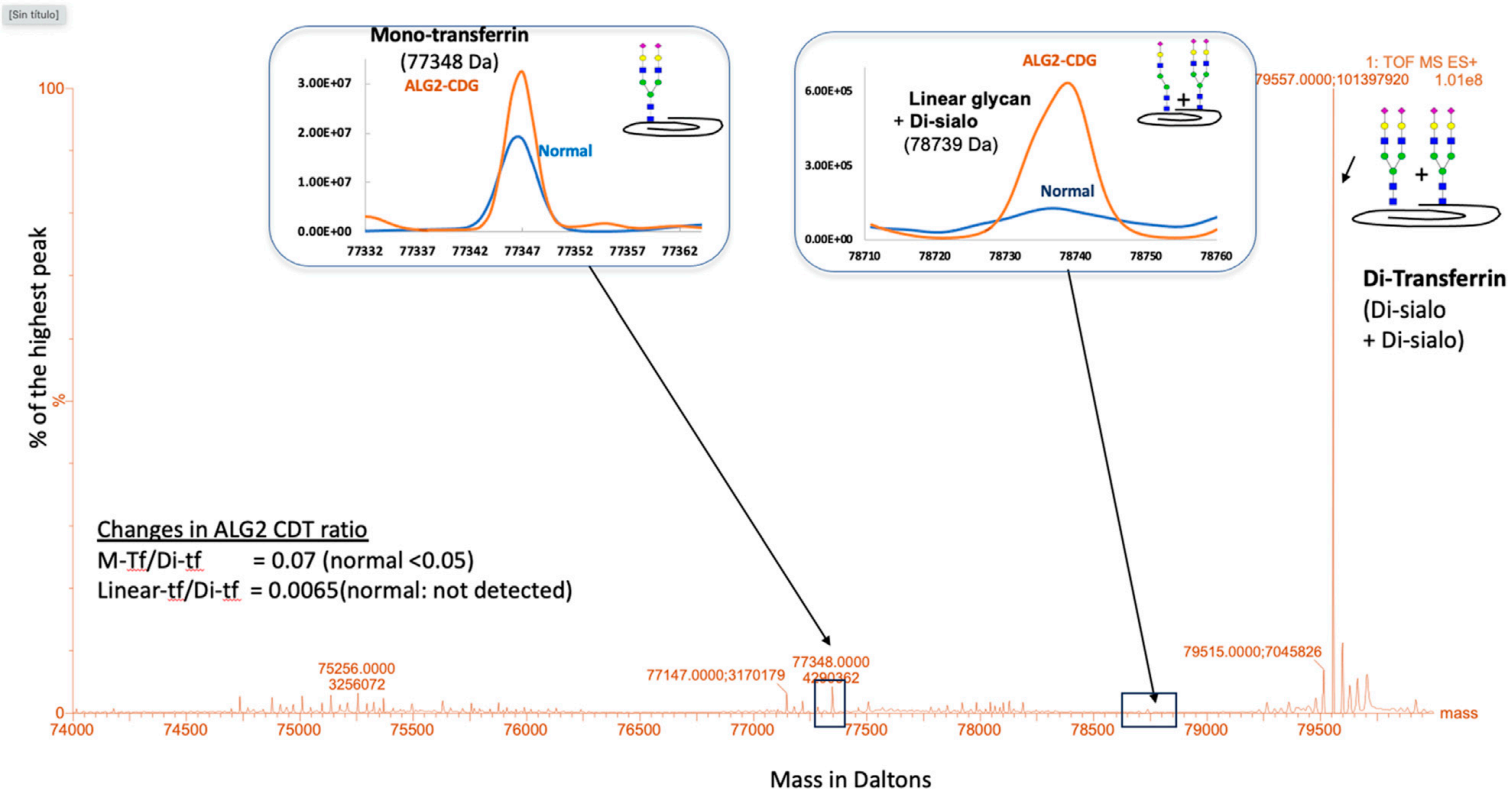
ESI-MS analysis of plasma transferrin isoforms. A type 1 CDG profile was identified with increased ratio of mono-transferrin (M-tf) to di-transferrin (Di-tf). Also, a transferrin isoform bearing a linear heptasaccharyde glycan (NeuAc-Gal-GlcNAc-Man2-GlcNAc2; linear-tf) was identified.

The carbohydrate-deficient transferrin (CDT) analysis is the primary biochemical test available for CDG diagnosis and was performed using accurate mass analysis of affinity purified transferrin protein using a LC-ESI-TOF MS system (Li et al., 2015). A mild type 1 CDG pattern was identified with an increased mono-oligosaccharide/di-oligosaccharide ratio. Also, an abnormal transferrin glycoform containing one linear complex heptasaccharide glycan (NeuAc-Gal-GlcNAc-Man2-GlcNAc2) and one normal disialo glycan was found. Interestingly, this unusual glycan has also been recently reported in Argentinian ALG2-CDG patients (Papazoglu et al., 2021) (Figure 2). To determine its presence in other plasma glycoproteins, IgG and transferrin were removed from plasma and the remaining fraction tested, both the linear and fucosylated linear glycans were found to be increased (Table 1), suggesting this is likely a generalized glycosylation abnormality in ALG2-CDG.

**TABLE 1:** N-glycan changes in child’s plasma. * Predicted structure and component are shown. Simplified structure of each N-glycan is shown as pictures: lavender diamond represents sialic acid (Neu5Ac), yellow circle represents galactose (gal), green circle represents mannose (Man), red triangle represents fucose (Fic) and dark blue square represents N-acetyl-glucosamine (GlcNAc). Table: N-glycan changes in ALG2-CDG patient.

Plasma N-glycans (%total)		Proband	Reference normal (n = 60)	
*Predicted structure	Predicted glycan components		LOW	HIGH
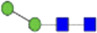	Man_2_GlcNAc_2_	0.18	*0.03*	*0.24*
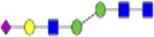	Neu5Ac_1_Gal_1_Man_2_GlcNAc_3_ (Linear glycan)	**1.80**	*0.06*	*1.48*
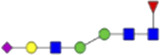	Fuc_1_Neu5Ac_1_Gal_1_Man_2_GlcNAc_3_ (Linear glycan - fuc)	**0.22**	*0.00*	*0.21*
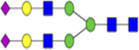	Neu5Ac_2_Gal_2_Man_3_GlcNAc_4_ (Disialo glycan)	49.68	*25.55*	*55.21*

	Transferrin N-glycan (%total)	Proband	Reference normal (n = 60)	
			LOW	HIGH
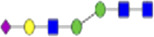	Neu5Ac_1_Gal_1_Man_2_GlcNAc_3_ (Linear glycan)	**1.26**	*0.58*	*1.17*
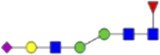	Fuc_1_ Neu5Ac_1_Gal_1_Man_2_GlcNAc_3_ (Linear glycan - fuc)	**0.10**	*0.00*	*0.10*
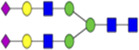	Neu5Ac_2_Gal_2_Man_3_GlcNAc_4_ (Disialo glycan)	78.76	*61.04*	*79.42*

	Tf and IgG depleted plasma fraction N-glycan (%total)	Proband	Reference normal (n = 60)	
			LOW	HIGH
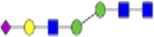	Neu5Ac_1_Gal_1_Man_2_GlcNAc_3_ (Linear glycan)	**1.79**	*0.53*	*1.00*
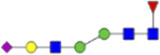	Fuc_1_Neu5Ac_1_Gal1Man_2_GlcNAc_3_ (Linear glycan - fuc)	**0.17**	*0.00*	*0.12*
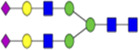	Neu5Ac_2_Gal_2_Man_3_GlcNAc_4_ (Disialo glycan)	56.68	*39.9*	*60.7*

Bold values represents the abnormal values.

Italic values represents the percentage (%).

**FIGURE 3:**
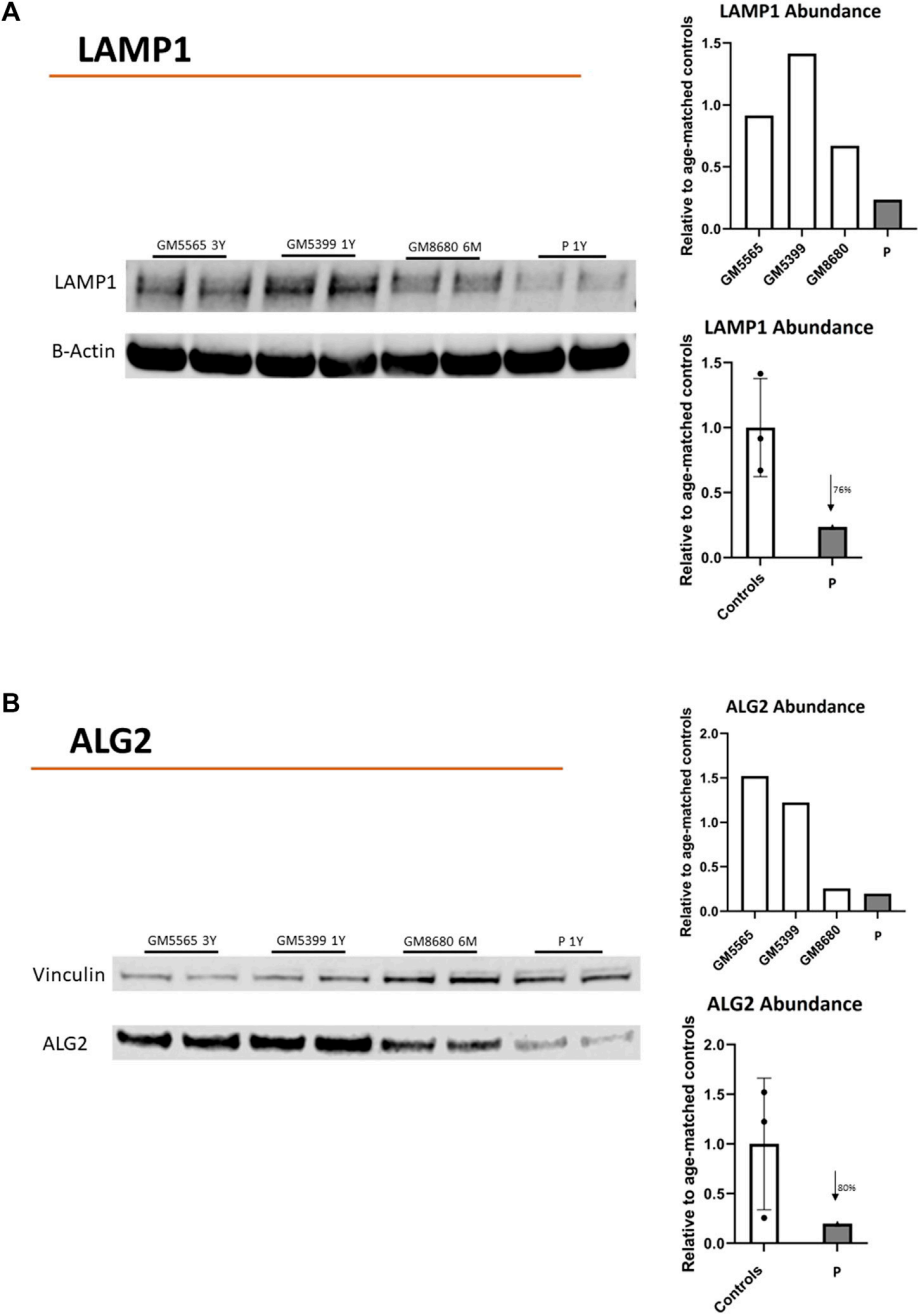
Protein abundance of LAMP1 and ALG2. Immunoblots showing LAMP-1 **(A)** or ALG2 **(B)** protein abundance in childs’ fibroblasts (P). Healthy Controls (GM5565, 3-year-old; GM5399, 1 year-old; GM8680 6 months-old). Beta-Actin or vinculin were used as a loading control. LAMP-1, ALG2, B-ACTIN or VINCULIN bands in the blots were quantified using Li-Cor Odyssey Image Studio (version 3.1). The quantified values for LAMP1 or ALG2 were expressed relative to quantified value of BACT or VINCULIN, respectively. The average value (relative to BACT or VINCULIN) of all controls (in duplicate) were used to express the fold change of expression for child’s values (in duplicate). LAMP1: reduced 76% vs. control average. ALG2: reduced 80% vs. control average.

Additionally, we evaluated the presence of ALG2 and LAMP1 in the child’s fibroblasts. LAMP1 is a common marker of cellular glycosylation that has been previously shown to decrease in CDG fibroblasts (Ligezka et al., 2023). LAMP1 abundance was decreased by 76% compared to control cells (Figure 3A). The abundance of ALG2 was also reduced 80% compared to control cells (Figure 3B).

## 3 Discussion

This report provides a detailed analysis of the clinical and molecular characteristics of the first child of Mexican ancestry diagnosed with ALG2-CDG, an extremely rare autosomal recessive disorder, of which only 14 cases have been documented in the literature to date (Table 2). Consistent with previous cases, the child exhibits a congenital myasthenic syndrome manifesting with muscular weakness, perinatal asphyxia, and feeding-related complications stemming from suction issues. Additionally, the fatigue experienced during the feeding sessions led to inadequate nutrition. Neurological manifestations were also observed, including seizures, global developmental delay, hypotonia, and structural brain alterations in agreement with previous ALG2-CDG reports (Thiel et al., 2003; Cossins et al., 2013; Monies et al., 2014; Monies et al., 2016; Papazoglu et al., 2021; Ehrstedt et al., 2022). Also, no dystonia, facial weakness or hearing loss reported in some ALG2-CDG patients was identified in the child (Table 2).

**TABLE 2:** Clinical features of ALG2-CDG patients^a^.

Main symptoms	Reported cases	This report	Total	Ref.
ALG2-CDG	14/14	+	15/15	Thiel et al. (2003), Cossins et al. (2013), Monies et al. (2014), Monies et al. (2016), Papazoglu et al. (2021), Ehrstedt et al. (2022)
Intellectual disability/global developmental delay	10/14	+	11/15	Thiel et al. (2003), Cossins et al. (2013), Monies et al. (2014), Papazoglu et al. (2021), Ehrstedt et al. (2022)
Seizures	4/14	+	5/15	Thiel et al. (2003), Papazoglu et al. (2021)
Hypotonia	9/14	+	10/15	Thiel et al. (2003), Cossins et al. (2013), Monies et al. (2014), Papazoglu et al. (2021), Ehrstedt et al. (2022)
Brain structural alterations	4/14	+	5/15	Thiel et al. (2003), Papazoglu et al. (2021)
Microcephaly	3/14	+	4/15	Papazoglu et al. (2021)
Ocular symptoms (coloboma, strabismus, nystagmus and not specified)	2/14	+	3/15	Thiel et al. (2003), Papazoglu et al. (2021)
Predominantly proximal muscle weakness	10/14	+	11/15	Cossins et al. (2013), Monies et al. (2014), Monies et al. (2016), Ehrstedt et al. (2022)
Dystonia	1/14	-	1/15	Papazoglu et al. (2021)
Joint laxity	5/14	+	6/15	Cossins et al. (2013), Papazoglu et al. (2021)
Facial weakness	4/14	-	4/15	Cossins et al. (2013)
Hearing impairment	3/14	-	3/15	Papazoglu et al. (2021)
Gastrointestinal disorders (difficulty feeding, gastroesophageal reflux, diarrhea or hepatomegaly)	5/14	+	6/15	Thiel et al. (2003), Papazoglu et al. (2021), Ehrstedt et al. (2022)
Facial dysmorphism	2/14	+	3/15	Papazoglu et al. (2021)

^a^+, present; −, absent.

Dysmorphic facial features were identified in the child, including a prominent forehead, metopic prominence, fan-shaped eyebrows, long eyelashes, sunken eyes, full cheeks, thick nasal tip, wide nasal bridge, prominent upper lip, high palate, retrognathia, pointed ears with hypoplastic helix and prominent concha (Figure 1). It is important to note that, until now, facial dysmorphia has only been described in two previous cases, characterized by upward-slanting palpebral fissures, wide nasal bridge, and bilateral epicanthic folds (Papazoglu et al., 2021). Therefore, dysmorphic facial characteristics emerge as an additional distinctive feature of ALG2-CDG, emphasizing the importance of considering this phenotypic variability in future reports and studies on the disease.

Both the identified VUS c.964C>A p.(Pro322Thr) as well as the novel variant c.1055_1056 delinsTGA p.(Ser352Leufs*3) are predicted to be pathogenic through different bioinformatic tools. Additionally, the Pro322Thr variant has a low frequency in the population and is carried in a heterozygous state, also suggesting pathogenicity. In regard to the c.1055_1056 delinsTGA p.(Ser352Leufs*3) novel variant, it was absent from population databases and its characteristics support its classification as “likely pathogenic” according to ACMG standard and guidelines, considering criteria PVS1 and PM2 (Richards et al., 2015).

The CDT and plasma N-glycan analysis revealed the presence of a linear heptasaccharide glycan (NeuAc-Gal-GlcNAc-Man2-GlcNAc2) recently reported in Argentinian ALG2-CDG patients, further confirming the diagnosis and the relevance of this biomarker as specific for ALG2-CDG. This linear heptasaccharide would result because of impaired ALG2 function causing a deficiency in the branching of the biantennary N-glycan during lipid-linked oligosaccharide synthesis, with only one branch being fully synthesized. The transference of truncated glycans from the lipid-linked oligosaccharide to nascent *N*-glycoproteins during the first stage of *N*-glycan synthesis has been identified in other CDGs. For example, a tetrasaccharide biomarker has been identified for the diagnosis of ALG1-CDG (Bengtson et al., 2016; Zhang et al., 2016; González-Domínguez et al., 2021).

Deficient glycosylation was further confirmed with a substantial decrease in the expression of LAMP1, a cellular marker associated with glycosylation (Figure 3). Also, the decreased abundance of ALG2 is consistent with the predicted instability of the mutated proteins establishing the connection between altered glycosylation, reduced ALG2 abundance, and the observed clinical phenotype.

The integration of clinical, biochemical, and genetic findings reinforces the association between the identified genetic variants in ALG2 and the specific clinical presentation of this ALG2-CDG case. In the future, detailed phenotypic reports of new cases and the identification of additional pathogenic mutations will contribute to defining the spectrum and progression of the disease more precisely. These contributions will not only enhance the understanding of the syndrome but also facilitate the recognition of genotype-phenotype correlations for the various characteristics of this disorder.

## Data Availability

The raw data supporting the conclusion of this article will be made available by the authors, without undue reservation.
